# Effect of COX-2 inhibition on tendon-to-bone healing and PGE2 concentration after anterior cruciate ligament reconstruction

**DOI:** 10.1186/s40001-017-0297-2

**Published:** 2018-01-05

**Authors:** Martin Sauerschnig, Josef Stolberg-Stolberg, Carmen Schmidt, Valerie Wienerroither, Michael Plecko, Karin Schlichting, Carsten Perka, Christian Dynybil

**Affiliations:** 1 Center for Musculoskeletal Surgery, University Hospital Charité, Charitéplatz 1, 10117 Berlin, Germany; 2 Department of Experimental Trauma Surgery, Technical University of Munich, Ismaninger Straße 22, 81675 Munich, Germany; 3 Department of Orthopaedic Sports Medicine, Technical University of Munich, Ismaninger Straße 22, 81675 Munich, Germany; 4 Department of Trauma-, Hand- and Reconstructive Surgery, University Hospital Muenster, Albert-Schweitzer-Campus 1, 48149 Münster, Germany; 5 Trauma Hospital Graz, Unfallkrankenhaus der Allgemeinen Unfallversicherungsanstalt (AUVA), Göstinger Straße 24, 8020 Graz, Austria

**Keywords:** Anterior cruciate ligament, Tendon-to-bone healing, Prostaglandin E2, Cyclooxygenase-2 inhibitor

## Abstract

**Background:**

Non-steroidal anti-inflammatory drugs are commonly used to reduce pain and inflammation in orthopaedic patients. Selective cyclooxygenase-2 (COX-2) inhibitors have been developed to minimize drug-specific side effects. However, they are suspected to impair both bone and tendon healing. The objective of this study is to evaluate the effect of COX-2 inhibitor administration on tendon-to-bone healing and prostaglandin E (PGE2) concentration.

**Methods:**

Thirty-two New Zealand white rabbits underwent reconstructions of the anterior cruciate ligaments and were randomized into four groups: Two groups postoperatively received a selective COX-2 inhibitor (Celecoxib) on a daily basis for 3 weeks, the two other groups received no postoperative COX-2 inhibitors at all and were examined after three or 6 weeks. The PGE2 concentration of the synovial fluid, the osseous integration of the tendon graft at tunnel aperture and midtunnel section, as well as the stability of the tendon graft were examined via biomechanic testing.

**Results:**

After 3 weeks, the PGE2 content of the synovial fluid in the COX-2 inhibitor recipients was significantly lower than that of the control group (*p* = 0.018). At the same time, the COX-2 inhibitor recipients had a significantly lower bone density and lower amount of new bone formation than the control group (*p* = 0.020; *p* = 0.028) in the tunnel aperture. At the 6-week examination, there was a significant increase in the PGE2 content within synovial fluid of the COX-2 inhibitor recipients (*p* = 0.022), whose treatment with COX-2 inhibitors had ended 3 weeks earlier; in contrast, the transplant stability decreased and was reduced by 37% compared to the controls.

**Conclusions:**

Selective COX-2 inhibitors cause impaired tendon-to-bone healing, weaken mechanical stability and decrease PGE2 content of the synovial fluid. The present study suggests a reluctant use of COX-2 inhibitors when tendon-to-bone healing is intended.

## Background

Anterior cruciate ligament (ACL) rupture is a rather frequent injury among people who engage in sports or leisure activities, with an incidence of 0.8 per 1000 among the general population [1]. Among operative treatment options, ligament reconstruction with high primary graft stability is eligible to prevent malfunction and potentially postpone consequent osteoarthritis [2–5].

However, postoperative ACL graft rupture and instability is one of the leading causes for revision surgery [6, 7]. Hence, it is essential to understand basic factors affecting the healing process at the interface between tendon and bone to improve treatment and provide long-term graft durability.

Non-steroidal anti-inflammatory drugs (NSAIDs) are successfully used for pain alleviation in orthopaedic patients. Inhibition of the enzymes cyclooxygenase-1 and -2 (COX-1, COX-2) prevents synthesis of the inflammatory mediators such as prostaglandin E2 (PGE2) [8, 9]. However, there exists uncertain evidence for impaired fracture and tendon healing by both selective and unselective COX inhibitors [10–14]. PGE2 as downstream product is heavily involved in bone homeostasis [15, 16]. In rats treated with selective COX-2 inhibitors, the endogenous PGE2 synthesis was reduced and fracture healing was disturbed, just as in COX-2^−/−^ mice [17, 18]. Contrariwise, direct prostaglandin administration causes hyperostosis and an increase in trabecular and cortical mass [19–22]. Similarly, tendon healing involves inflammatory processes including PGE2 release [23, 24]. Here, literature describes both degenerative morphologic changes and superior biomechanical properties after direct prostaglandin application [25–27].

Little is known about the effect of COX inhibitors on tendon-to-bone integration [28]. Rotator cuff, achilles, and patella tendon repairs in rats show reduced pull-out strength and inconsistent fibrocartilage regrowth after parecoxib treatment [11, 25, 29]. Osteoclast inhibition improves ACL repair in rabbits [30]. However, the influence of a selective COX-2 inhibitor treatment on intraarticular PGE2 concentration and the effect on tendon-to-bone healing process has not yet been studied. The objective of the present work is to correlate synovial PGE2 concentration after COX-2 inhibitor treatment to osseous tendon graft integration as well as to graft stability after ACL reconstruction.

## Methods

### Study design

ACL reconstruction was performed using autologous semitendinosus tendon graft on 32 skeletally mature female New Zealand white rabbits (3.5 ± 0.2 kg body weight). The animals were randomized into four groups: Two groups were examined 3 weeks after surgery, with one group being administered selective COX-2 inhibitor (Celecoxib) on a daily basis (3 weeks COX-2 inhibitor group), while the other group received no COX-2 inhibitor (3 weeks control group). The third group (6 weeks COX-2 inhibitor group) received COX-2 inhibitor on a daily basis over 3 weeks postoperatively (like 3 weeks COX-2 inhibitor group) and no COX-2 inhibitor for further 3 weeks and was examined 6 weeks after ACL reconstruction. The fourth group received no COX-2 inhibitor during the entire 6 weeks (6 weeks control group). Celecoxib was diluted in a 5-ml syringe with drinking water in a weight-adapted concentration (10 mg/kg body weight per day) and was administered immediately under control of complete ingestion. The dose of Celecoxib was chosen based on the dose known to inhibit rotator cuff tendon-to-bone and fracture healing and corresponded approximately to the twofold dose of the assigned maximum dose for humans (Pfizer, Inc., New York, NY, USA) [29, 31]. Eventually, animals were anesthetized with sodium thiopental and sacrificed utilizing an overdose of potassium chloride.

### Operation procedures

**Fig. 1:**
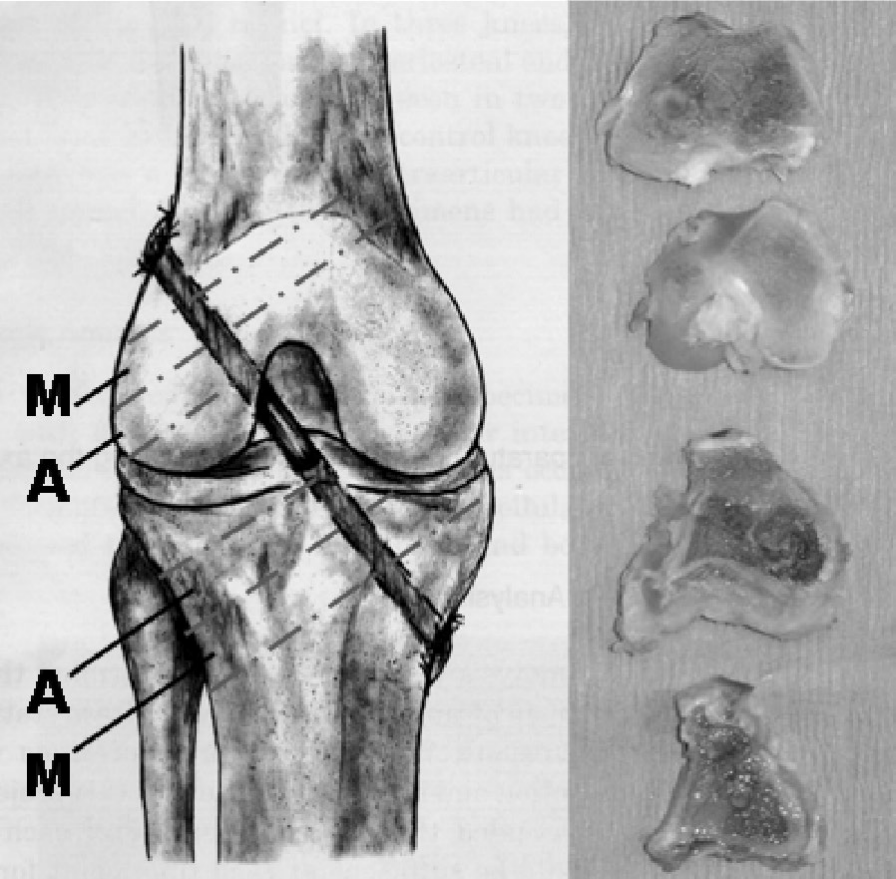
The bone tunnel was subdivided as a tunnel aperture (A) and mid-tunnel (M) sections

Rabbits were anesthetized using intraperitoneal injection of ketamine (20 mg/kg) and medetomidine hydrochloride (0.25 mg/kg). Anesthesia was maintained using 2.5% Isoflurane. Animals were placed in supine position and surgery was performed using standardized sterile technique. The knee joint was medially arthrotomized and the ACL was resected. The semitendinosus tendon was harvested from the ipsilateral knee. The graft was placed under tension at the anatomical ACL attachment sites through 2.2-mm diameter bone tunnels in the lateral femoral condyle and proximal tibia. The tibial tunnel was approximately 12 mm and the femoral tunnel approximately 10 mm in length. The graft was pretensioned manually with 5 N and was then secured at the periosteum and surrounding soft tissues using 4-0 Ethibond suture with the knee maintained at 60° flexion according to the protocol of Rodeo et al. [30] (Fig. 1). The wound was closed in layers. Buprenorphine (0.075 mg/kg) was administered subcutaneously for analgesia during the postoperative period every 8 h until apparent wound healing and weightbearing activities were permitted ad libitum. All procedures were authorized by the State Office for Occupational Safety, Health Protection, and Technical Safety Berlin (LAGetSi; Project number: G0028/06). This study was carried out in strict accordance with the recommendations in the Guide for the Care and Use of Laboratory Animals of the National Institutes of Health. All surgery was performed under general anesthesia, and all efforts were made to minimize suffering.

### Macroscopic transplant assessment

**Fig. 2:**
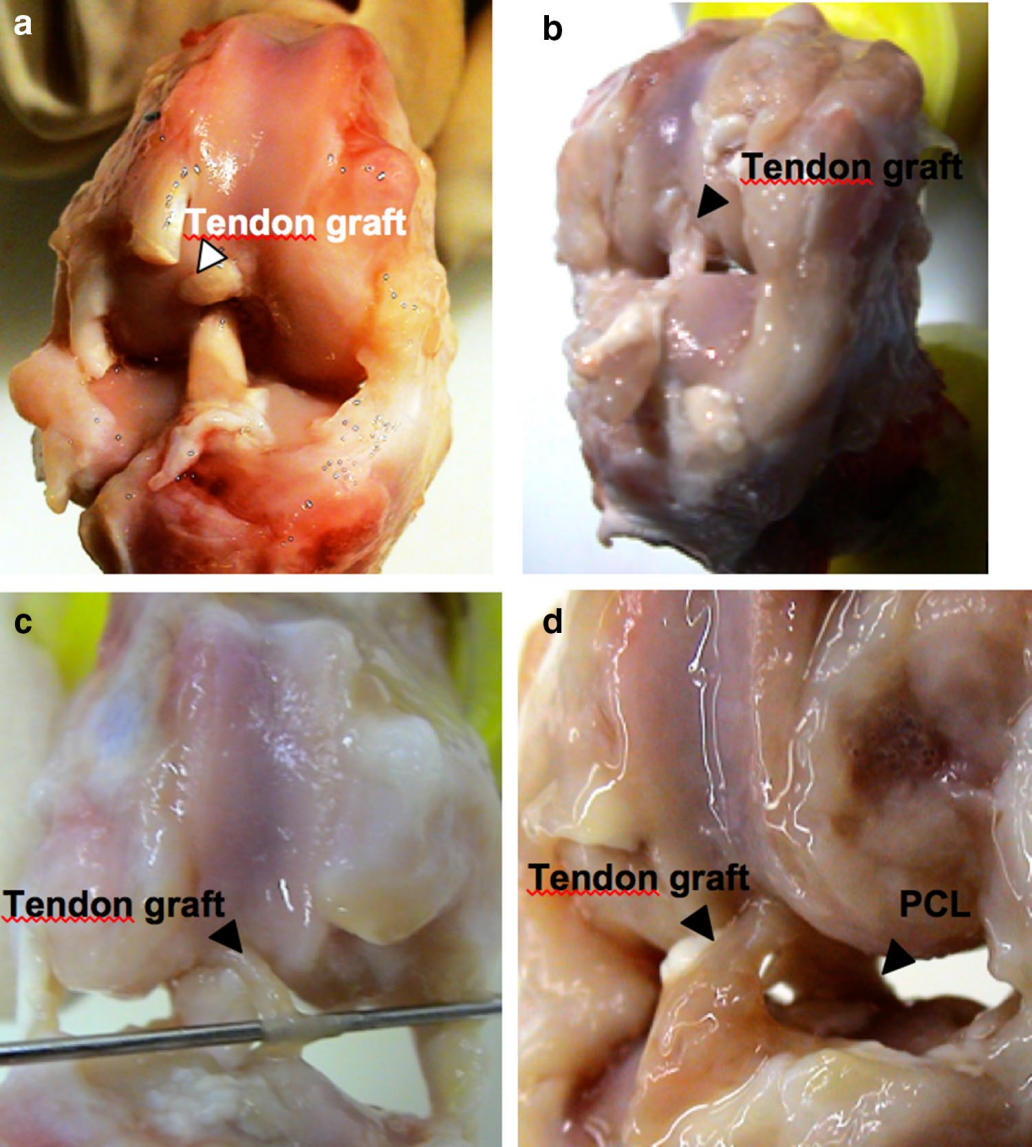
At the time point of examination, the tendon graft condition was classified morphologically as follows: **a** grade I—good condition (no atrophy, clearly demarcated from the rest of the joint structures); **b** grade II—atrophic (decrease of the transplant diameter); **c** grade IIIa—distinctly atrophic (clear decrease of the transplant diameter, transparent); **d** grade IIIb—distinctly atrophic and in part highly covered fibrinously, difficult to delimit from the surrounding soft tissues)

29 of 32 animals were macroscopically assessed with regard to the condition of the transplant. One rabbit was prematurely euthanized due to fibrinous tracheitis (6 weeks COX-2 group) and two further animals were excluded from the study after autoaggressive behavior and subsequent wound infection (3 weeks control group). The graft condition was classified morphologically as follows: (I) good condition (no atrophy, clearly demarcated from the rest of the joint structures; Fig. 2a), (II) atrophic (decrease of the transplant diameter; Fig. 2b), (IIIa) distinctly atrophic (clear decrease of the transplant diameter, transparent; Fig. 2c), (IIIb) distinctly atrophic and in part highly covered fibrinously (difficult to delimit from the surrounding soft tissues Fig. 2d).

### PGE2 enzyme immunoassay

Analysis of PGE2 concentration of the synovial fluid from the operated knee joints was possible in 21 animals [5 (3 weeks COX-2); 5 (3 weeks control); 6 (6 weeks COX-2); 5 (6 weeks control)]. Aspiration was performed by a veterinary technician utilizing a syringe via a standardized portal just medial to the patellar tendon. A sufficient quantity could not be aspirated from the remaining eight animals. Samples were harvested instantaneously after euthanasia, stored at – 80 °C and thawed at room temperature for testing. The concentration of synovial PGE2 was measured in double examinations using the Sandwich ELISA (Enzyme-Linked Immunoabsorbent Assay) method (R & D Systems, Minneapolis, MN, USA). The sensitivity of this PGE2 assay is 13.4 pg/ml.

### Transplant stability

Biomechanical testing was carried out in 20 animals [5 (3 weeks COX-2); 4 (3 weeks control); 6 (6 weeks COX-2); 5 (6 weeks control)]. Nine animals could not be examined due to adhesion of the tendon graft with the posterior cruciate ligament or pronounced atrophy of the tendon graft. The operated legs were exarticulated in the hip joint and stored at − 20 °C. Twelve hours before the examination, they were thawed at 4 °C and kept moist with physiologic saline solution during the entire procedure. The femoral and tibial bone ends were fixed with polymethylmethacrylate in aluminum cylinders and cleaned of all remaining soft tissue. They were mounted with the aluminum cylinder in a material test machine (Zwick-1455; Zwick GmbH, Ulm, Germany) and the tendon graft was carefully aligned along the axis of traction. The sutures at the periosteum were abscised and the construct was then stressed with a test speed of 1 mm/s until failure occurred. Maximum load to failure and ultimate construct stiffness were recorded. The failure mode was protocolled according to the following categories: graft rupture at femoral or tibial insertion site, midsubstance rupture, osseous avulsion or femoral or tibial graft pull-out.

### New bone formation and bone density

**Fig. 3:**
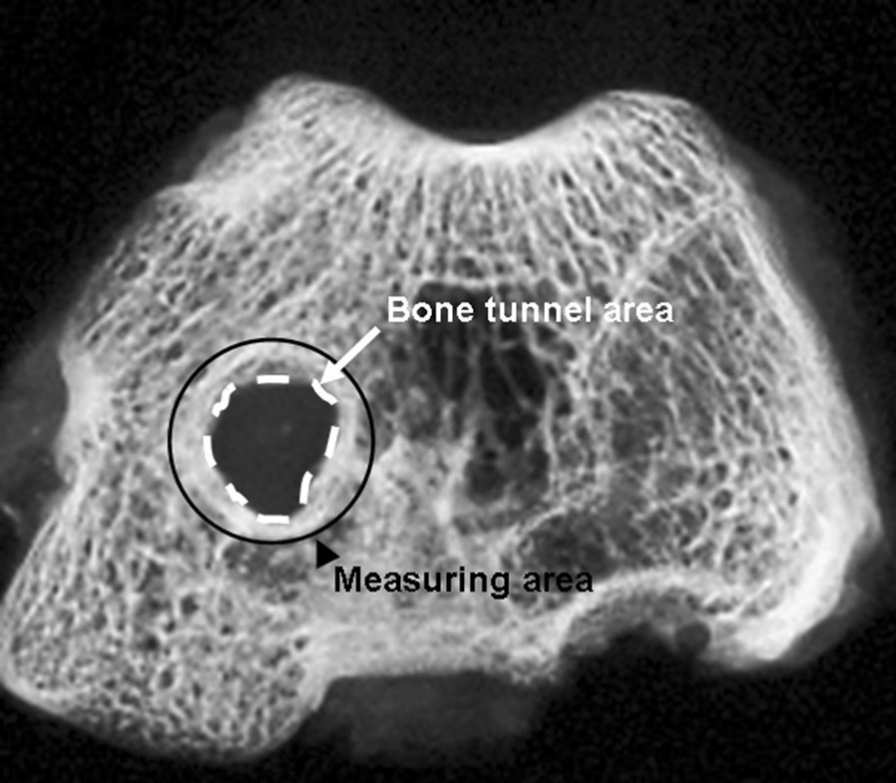
The bone density and the bone area were examined in a defined measurement area around the bone tunnel in the pQCT

**Fig. 4:**
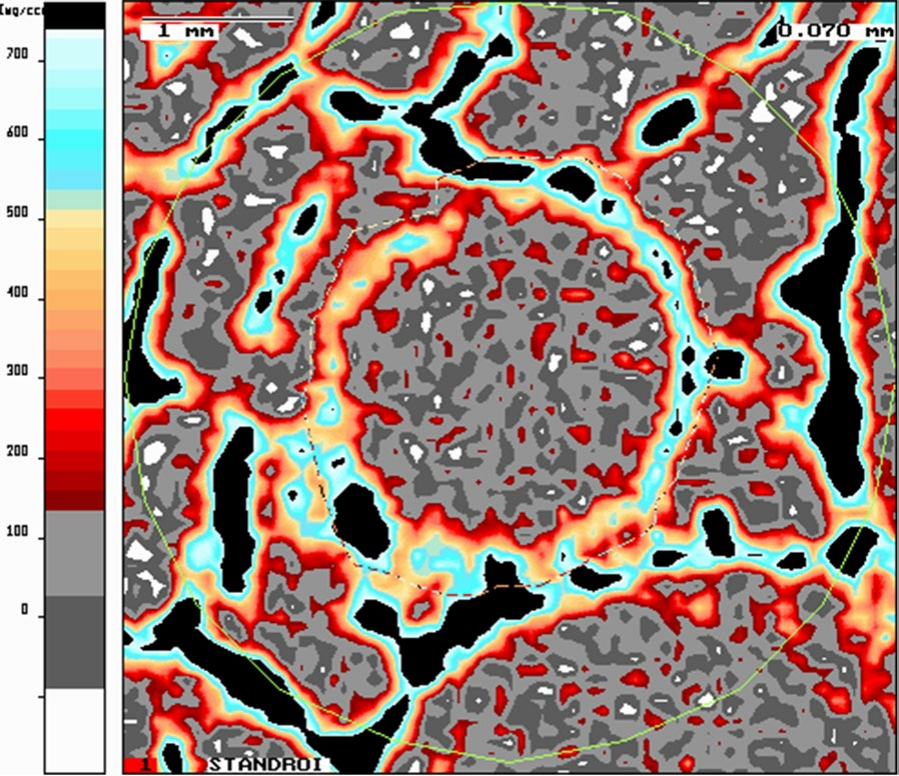
The various bone densities in the bone surrounding the tendon graft were morphologically assessed in the pQCT imaging by means of a color scale

Tibial and femoral bone tunnel lengths were measured and two sections were analyzed utilizing peripheral quantitative computed tomography pQCT as follows: The section near the joint line was defined as the tunnel aperture [A] and the middle bone tunnel section was defined as the midtunnel section [M]. They were both sawed into equal bone slices with a mean thickness of about 3.3 mm (femoral) and 4 mm (tibial) perpendicular to the bone tunnel direction using a precision saw system (Exakt; Apparatebau GmbH, Norderstedt, Germany) (Fig. 1). The bone slices were then mounted axially in an in vivo high-resolution peripheral quantitative computed tomography (pQCT) scanner (XCT Research SA; StraTec Medizintechnik; Pforzheim, Germany). The measurement area was determined with computer assistance according to a standardized procedure (Fig. 3). The mean bone density (hydroxyapatite [HA] in mg/cm^3^) and the area of the newly formed bone (mm^2^) were examined in each section. The measuring threshold was set at 180 mg HA/cm^3^ for newly formed bone of trabecular and cortical density. Pre-existing trabecular bone was expected about 180 mg/cm^3^, pre-existing cortical bone at a density of at least 600 mg/cm^3^ [32–34]. For bone of subcortical density that was not yet completely mineralized (“*immature woven bone*”), the measurements were also performed at a threshold set of 100 mg HA/cm^3^ [35, 36]. The thresholds were chosen to be sure that no graft or other soft tissue was detected as “newly formed bone.” According to our measurements, the graft was the densest soft tissue structure in the standardized measurement area and had a mean density of 77.1 ± 3.1 mg/cm^3^. The interassay coefficient of variation for these measurements was below 4%. A standard phantom measurement was performed on a daily basis with three measurement values within the given reference ranges provided by the manufacturer. Besides the quantitative analysis of the mean bone density and the new bone formation, the various bone densities in the bone surrounding the tendon graft were morphologically assessed with the help of the pQCT imaging with a color scale (Fig. 4). The voxel size for the examinations was 70 µm. Altogether, pQCT examinations could be carried out in 28 animals. In one animal, a clear identification of the bone tunnel in the pQCT was not possible.

### Statistical evaluation

The PGE2 content of the synovial fluid, the area of the newly formed bone, the bone density in the bone tunnel, the maximal load to failure and the stiffness of the tendon graft were compared between the individual examination groups using the Wilcoxon–Mann–Whitney test. Differences between the bone tunnel sections at the tunnel aperture and at the midtunnel section in the same animal were calculated non-parametrically with the Wilcoxon rank-sum test for two related samples. Relationships of variables of newly formed bone area, bone density, PGE2 concentration of the synovial fluid, maximal load to failure or stiffness of the tendon graft were each ascertained according to the Spearman-Rho correlation coefficients. The significance level was defined as *p* < 0.05 among explorative analysis. The statistical evaluation was performed using the software program SPSS 17 (Statistical Package for Social Sciences, Inc.; Chigaco, IL; USA).

## Results

### PGE2 enzyme immunoassay

**Fig. 5:**
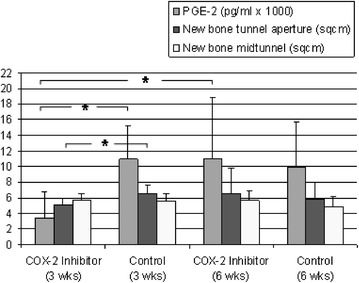
The synovial PGE2 concentration was significantly reduced after 3 weeks application of COX-2 inhibitor and was significantly increased 3 weeks after the last administration of COX-2 inhibitor in comparison to the 3-week examination time point. An increased new bone formation was temporally associated with an increased PGE2 concentration in the bone tunnel area near the joint area

After 3 weeks of COX-2 inhibitor administration, the PGE2 concentration of the synovial fluid was around one-third compared to the control group (3399.7 ± 3492.8 pg/ml vs. 10,871.2 ± 4274.9 pg/ml) and was therefore significantly lower (*p* = 0.018). At the 6 weeks time point (i.e., after a 3-week discontinuation of COX-2 inhibitor), the PGE2 content of the synovial fluid in the COX-2 inhibitor group had tripled and thus increased significantly to 11,098.7 ± 7738.2 pg/ml (*p* = 0.022). In the control group, the PGE2 value decreased around 10% (9920.7 ± 5815.42 pg/ml; Fig. 5; Table 1).

**Table 1:** Overview of the examination results regarding the mean, the standard deviation, and the statistical calculations

Investigation group	COX-2 inhibitor 3 weeks	Control 3 weeks	COX-2 inhibitor 6 weeks	Control 6 weeks
PGE-2 concentration in synovial fluid (pg/ml)	3399.7 (± 3492.8)	10,871.2 (± 4274.9)	11,098.7 (± 7738.2)	9920.7 (± 5815.4)
	*p = 0.018		°*p* = 0.667	
3 wks vs 6 wks	*p* = 0.022	*p* = 0.921		
New bone area (mm^2^)	5.09 (± 0.98)	6.45 (± 1.23)	6.55 (± 3.2)	5.7 (± 2.39)
A	*p = 0.028		°*p* = 0.699	
3 wks vs 6 wks	*p* = 0.302	*p* = 0.439		
New bone area (mm^2^)	5.63 (± 0.98)	5.55 (± 1.02)	5.59 (± 1.34)	4.75 (± 1.4)
M	*p = 0.897		°*p* = 0.317	
3 wks vs 6 wks	*p* = 0.699	*p* = 0.317		
New bone area (mm^2^)				
A vs M	*p* = 0.123	*p* = 0.028	*p* = 0.345	*p* = 0.398
Bone mineral density (mg HA/mm^3^)	453.92 (± 41.85)	504.23 (± 33.77)	484.78 (± 63.61)	465.04 (± 77.91)
A	*p = 0.020		°*p* = 0.731	
3 wks vs 6 wks	*p* = 0.302	*p* = 0.332		
Bone mineral density (mg HA/mm^3^)	231.57 (± 31.81)	222.52 (± 33.31)	216.76 (± 46.18)	216.88 (± 40.25)
M	*p = 0.414		°*p* = 0.228	
3 wks vs 6 wks	*p* = 0.651	*p* = 0.475		
Bone mineral density (mg HA/mm^3^)				
A vs M	*p* = 0.012	*p* = 0.028	*p* = 0.028	*p* = 0.018
Failure load (N)	69.33 (± 50.47)	28.2 (± 20.89)	37.4 (± 16.82)	59.57 (± 53.6)
Failure load (N)	*p = 0.171		°*p* = 0.914	
3 wks vs 6 wks	*p* = 0.240	*p* = 0.394		
Stiffness (N/mm)	9.15 (± 5.64)	5.24 (± 5.47)	6.29 (± 3.79)	10.68 (± 10.21)
Stiffness (N/mm)	*p = 0.352		°*p* = 0.762	
3 wks vs 6 wks	*p* = 0.522	*p* = 0.522		

*A* tunnel aperture, *M* midtunnel, *wks*  weeks

* *p* = 3 wks COX-2 inhibitor vs 3 wks control

°*p* = 6 wks COX-2 inhibitor vs 6 wks control

### Macroscopic transplant condition

At the 3 weeks examination time point, 4 (50%) of the tendon grafts of the COX-2 inhibitor group were in good condition, partially hypertrophic, and well delimitable; 4 (50%) of the tendon grafts appeared slightly atrophic. At the same examination time point, around 3 (50%) of the tendon grafts in the control group were in good condition, 2 (33%) slightly atrophic, and 1 (17%) highly atrophic. At the 6-week time point in the COX-2 inhibitor group, 4 (57%) of the tendon grafts were in good condition, 3 (43%) were highly atrophic and difficult to identify. In the control group, 4 (50%) were in good condition, 2 (25%) slightly atrophic, and 2 (25%) highly atrophic (Table 2).

**Table 2:** Distribution of the macroscopically assessed transplant condition in the individual examination groups

Examination group	Good condition	Slightly atrophic	Distinctly atrophic
COX-2 inhibitor 3 weeks	*n* = 4	*n* = 4	*n* = 0
Control 3 weeks	*n* = 3	*n* = 2	*n* = 1
COX-2 inhibitor 6 weeks	*n* = 4	*n* = 0	*n* = 3
Control 6 weeks	*n* = 4	*n* = 2	*n* = 2

### pQCT

**Fig. 6:**
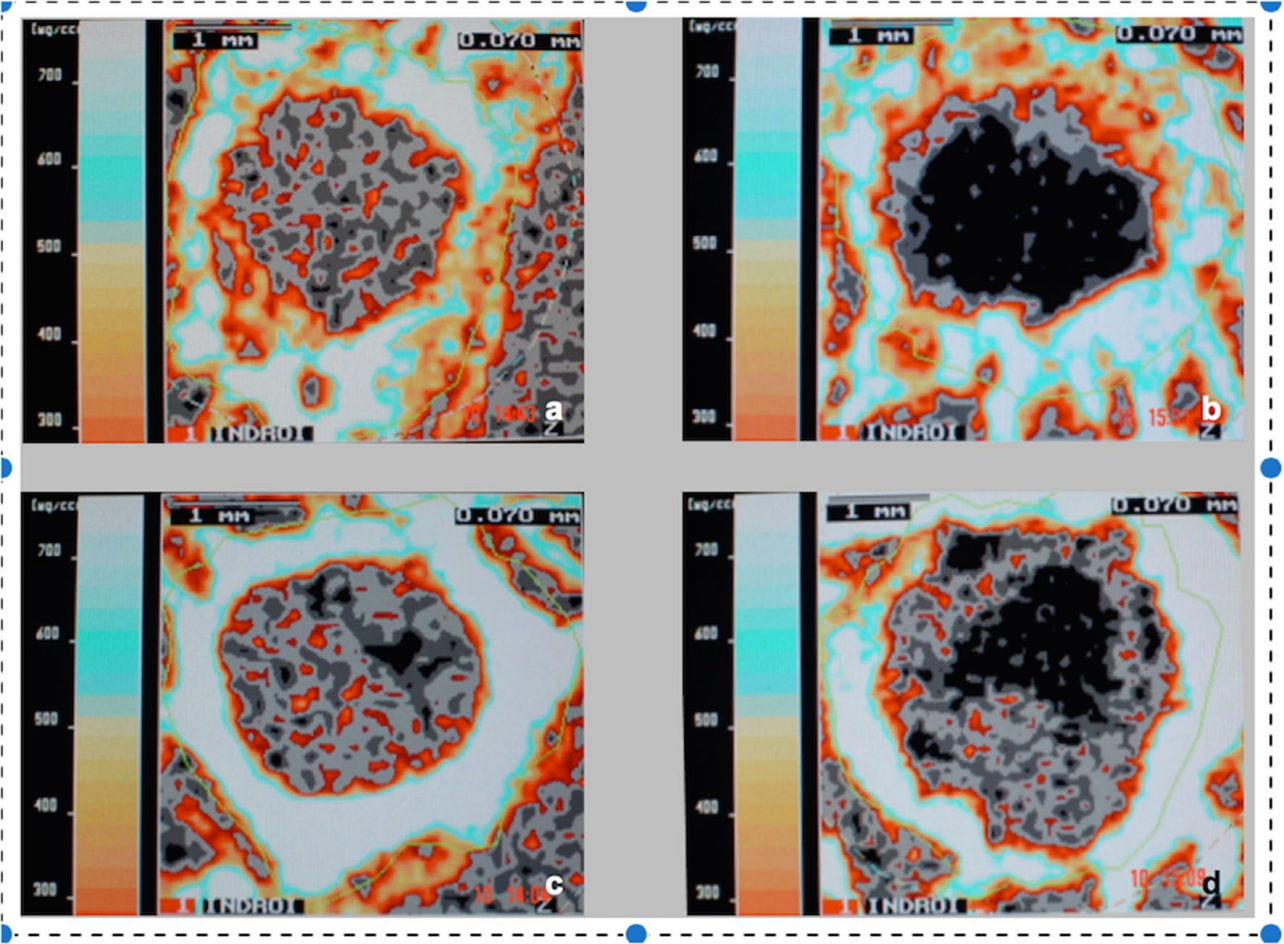
**a**, **b** After 3 weeks of treatment with COX-2 inhibitor, the bone area of cortical density (≥ 180 mg/cm^3^ HA) in the tunnel aperture section was significantly smaller than in the controls. **c**, **d** At the 6 weeks examination time point (i.e., 3 weeks after the last administration of COX-2 inhibitor), the cortical bone area in the COX-2 inhibitor group was larger. In the control group, a reduction of the cortical bone area was found

In all of the examination groups, a decrease of the bone density in the bone tunnel edge area was recognizable with increasing proximity to the tendon graft. Bone in direct proximity to the tendon graft had a density of less than 180 mg/cm^3^. Both in the COX-2 inhibitor group and in the control group, it was observed that the circularly arranged intermediate areas of lower density of the tendon graft mineralized over time. Simultaneously, the trabecular and cortical bone at the tunnel edge areas (Fig. 6a, b) that was still separated by bone of subtrabecular density (< 180 mg/cm^3^ HA) at the 3 weeks time point had merged into each other at the 6 weeks time point (Fig. 6c, d). After 3 weeks of COX-2 inhibitor treatment, the bone area of trabecular and cortical density (≥ 180 mg/cm^3^ HA) was significantly lower than in the controls 3.94 ± 0.87 vs 5.30 ± 1.03, respectively (*p* = 0.043), particularly in the femoral bone tunnel section near the joint line (*p* = 0.029; Fig. 6a, b). At the 6 weeks examination time point (i.e., 3 weeks after the last administration of the COX-2 inhibitor), the trabecular and cortical bone area in the same examination area in the COX-2 inhibitor group was larger. In the control group, a reduction of the bone area around the tendon graft was found (Fig. 6c, d).

### New bone formation

After 3 weeks of COX-2 inhibitor treatment, the bone area of subcortical density (< 180 mg/cm^3^ HA) as well as that of trabecular and cortical density (≥ 180 mg/cm^3^ HA) in the bone tunnel aperture section was significantly lower than in the controls (*p* = 0.028; *p* = 0.043). Comparing the new bone formation in the tunnel aperture and midtunnel area, a significantly stronger new bone formation was found in the tunnel aperture at the 3 weeks examination time point among controls (*p* = 0.028; Fig. 5; Table 1). After 6 weeks, there were no statistically significant differences, neither at the tunnel aperture nor in the midtunnel section. In the COX-2 inhibitor group, the newly formed bone area increased around 20%, in comparison to the 3-week examination time point in the tunnel aperture section (Fig. 5, Table 1). In contrast, the bone area of the controls was reduced in the tunnel aperture by about 11% and in the midtunnel by about 14%, in comparison to the 3-week examination time point (Fig. 5, Table 1). In the tunnel aperture, there was an increase in bone area in temporal association to an evident increase in PGE2 values, which were not statistically significant, respectively (Fig. 5, Table 1).

### Bone density

**Fig. 7:**
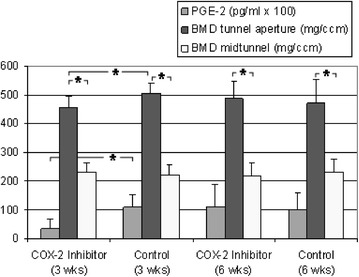
At the 3 weeks examination time point, the bone density in the tunnel aperture section was significantly reduced in the COX-2 inhibitor group in comparison to the controls. An increase in bone density in the tunnel aperture section was temporally associated with an increased PGE2 concentration. The bone density in the tunnel aperture section was significantly increased as compared to the mid-tunnel section in all of the examination groups

At the 3 weeks examination time point, the bone density in the tunnel aperture was significantly reduced in the COX-2 inhibitor group, in comparison to the controls (*p* = 0.020; Fig. 7, Table 1). At the 6 weeks examination time point (i.e., after 3 weeks COX-2 inhibitor discontinuation), the bone density in the tunnel aperture increased around 6%, with increased PGE2 concentration of the synovial fluid. The controls had their highest bone density in the tunnel aperture and highest PGE2 concentration of synovial fluid at the 3 weeks examination time point. Thus, the bone density, similarly to the new bone formation, correlated temporally with the concentration of PGE2, at least in the tunnel aperture. In the midtunnel, there were no significant differences between the groups, neither at the 3 weeks nor at the 6 weeks examination time point. The bone density was on average twice as high in the tunnel aperture as in the midtunnel section in all of the examination groups (*p* < 0.05).

### Transplant stability

**Fig. 8:**
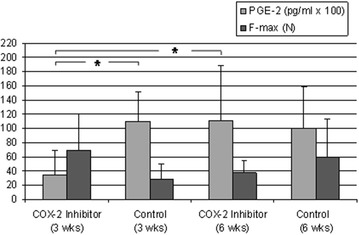
At the 6 weeks examination time point (i.e., 3 weeks after the last COX-2 inhibitor application), the failure load in the COX-2 inhibitor group was lower than it was at the 3 weeks examination time point. The failure load of the controls was higher after 6 weeks than it was at the 3-week examination time point, as well as compared to the COX-2 inhibitor group at the same examination time point. A decrease of the failure load of the tendon graft tended to correlate with an increase of the concentration of PGE2 (*p* = 0.056)

At the 3 weeks examination time point, the maximum failure load of the tendon graft in the COX-2 inhibitor group was elevated by around 60%, with significantly reduced PGE2 concentration in the synovial fluid compared to controls (COX-2 inhibitor group: 69.3 ± 50.5 N; control group: 28.2 ± 20.9 N; Fig. 8; Table 1). The resistance of the tendon graft against deformation in length direction (i.e., stiffness) was 9.2 ± 5.6 N/mm in the COX-2 inhibitor group after 3 weeks and therefore was elevated by around 43% compared to controls. At the 6 weeks examination time point (i.e., 3 weeks after the last COX-2 inhibitor application), the maximum failure load in the COX-2 inhibitor group was around 46% lower than at the 3-week examination time point (37.4 ± 16.8 N) and stood in temporal correlation with a significantly increased PGE2 concentration in comparison to the 3-week examination time point. The failure load of the controls was around 53% higher than at the 3 weeks examination time point (59.57 ± 53.6 N) with lower PGE2 concentration. The failure load of the COX-2 inhibitor group was thereby reduced around 37% in comparison to the controls at the 6 weeks examination time point. The stiffness of the tendon graft, at 6.3 ± 3.8 N/mm, was reduced around 31% in the COX-2 inhibitor group at the 6 weeks examination time point in comparison to the 3 weeks COX-2 inhibitor group, and around 41% in comparison to the controls at the same examination time point. There was a tendency towards correlation between an increase of the PGE2 concentration with a decrease of the failure load of the tendon graft but remained above the chosen level of significance (*r*^2^ = − 0.502; *p* = 0.056).

### Failure mode

In the 3 weeks COX-2 inhibitor group, the failure mode was an interosseous tendon graft failure in 5 (83.3%) of the cases, of which in two cases the central tendon graft part was partially pulled out off the peripheral tendon graft part that obviously remained connected with the bone tunnel (“degloving”). In one case (16.7%), a tendon graft rupture occurred in the joint space (midsubstance rupture). In the controls, the failure mode at the same examination time point was distributed equally between interosseous tendon graft extractions (*n* = 2) and ligamentary graft ruptures in the joint space (*n* = 2). After 6 weeks (i.e., 3 weeks after the last COX-2 inhibitor application), a partial interosseous tendon graft extraction occurred in 3 (75%) of the cases in the COX-2 inhibitor group and a complete tendon graft extraction in 1 (25%). The tendon graft tore ligamentarily within the bone tunnel in 3 controls (50%), and the other 3 (50%) had a ligamentary graft failure in the joint space.

## Discussion

The objective of the present study is to evaluate the effect of a selective COX-2 inhibitor on tendon-to-bone healing and PGE2 concentration in synovial fluid. Our findings indicate that COX-2 inhibition causes a significant decrease of PGE2 concentration in the synovial fluid that might lead to impaired bone formation and reduced graft stability over time.

Bone density as well as the newly formed bone area were significantly lower in the tunnel aperture after 3 weeks of COX-2 inhibitor administration compared to our control group. This is consistent with literature as enchondral ossification occurring during bone healing can be inhibited by Celecoxib [28]. Furthermore, COX-2^−/−^ mice show delayed fracture healing and selective COX-2 inhibitor application causes impaired bone healing in rats [17, 37]. However, the exact mechanism remains a matter of speculation: mesenchymal cell differentiation, neoangiogenesis controlled by PGE2 and impaired choncrocyte differentiation may play a pivotal role [18, 38–42]. Surprisingly, the failure load after 3 weeks COX-2 inhibitor administration was averagely more than twice as high compared to the control group. Additionally, an increase in newly formed bone as well as bone density in the observation period of 6 weeks had no effect on the transplant stability, indicating that graft healing or the ligamentary components might be decisive for transplant stability rather than newly formed bone.

Tendon healing in in vitro as well as in vivo studies shows inconsistent responses to NSAIDs [23–25, 28]. For selective COX-2 inhibitors, some authors describe no effect on tendon healing [43]. Others report the time of application as decisive factor. While early usage seems to inhibit tendon repair, selective COX-2 inhibitor treatment at later time points does not negatively affect tendon stability [44–46]. For example, Virchenko et al. report a damaging effect of injective Parecoxib application directly after Achilles tendon dissection. At later time points, an improvement of the mechanical properties was observed [46]. Using our data, we cannot show evidence for the effect of late COX-2 inhibitor application. However, in contrast to the current opinion, there was the tendency of increased mechanical load capacity after 3 weeks of COX-2 inhibitor treatment, although this was not statistical significant (*p* = 0.171). Furthermore, macroscopic tendon examination and failure mode indicate a favorable effect of early COX-2 inhibitor use (Table 2).

Tendon-to-bone healing after surgical ACL reconstruction will not recreate the four zones of an intact enthesis as scar tissue is formed [28]. Additionally, the bone tunnel is divided into healing at the tunnel aperture and intra-tunnel healing [47–49]. Recent in vitro data suggest a reduction of tenocyte viability and calcification markers after COX-2 inhibitor treatment [50]. Histology reveals only very limited data about inconsistent fibrocartilage regrowth and impaired collagen organization [13, 29]. However, recent technical advances suggest peripheral quantitative CT as promising to assess microarchitecture of joint pathologies [51]. Using this method, we were able to show a decrease of bone area of trabecular and cortical density and less new bone formation directly after Celecoxib treatment.

PGE2 is essential for bone homeostasis and fracture healing. Direct injection into bone fractures leads to an increase of bone mass [15–22]. Tendon injury is also followed by an inflammatory phase, but direct PGE2 application can cause both degeneration and consolidation [23–27]. In the present study, PGE2 concentration of the synovial fluid was examined and a significant decrease was recorded directly after 3 weeks of Celecoxib treatment. After another 3 weeks of COX-2 inhibitor absence, the PGE2 concentration was significantly increased again. Obviously, synovial fluid has contact to the tendon graft and magnetic resonance imaging has revealed fluid influx into the bone tunnels [52, 53]. Consistent with literature, there was a significant decrease of bone density and new bone formation during low PGE2 concentrations with temporal correlation and a slightly increased new bone formation and bone density at the 6 weeks time point. Other studies show no effect of early PGE2 application indicating that there is sufficient PGE2 during the inflammatory phase of callus formation and a drop off at later stages [54, 55]. The relationship between tendon biology and PGE2 remains unclear. Our study shows reduced graft stability after a PGE2 increase 6 weeks after surgery. In contrast, the control group showed an increase of graft stability with falling PGE2 levels, indicating the importance during the early healing phase. To our knowledge, there is no study evaluating the direct effect of PGE2 on tendon-to-bone healing [16, 28].

Limitations of the present study include graft and synovial fluid examination only at two time points. It is unknown whether graft stability, morphology, and PGE2 concentration will change during the following time course. However, failure modes indicate ligamentary components to be decisive at early time points while osseous integration happens to play a pivotal role at later time points only. Additionally, other cytokines might play a decisive role during graft healing [56].

## Conclusions

Concluding, literature shows contradicting evidence on the effect of NSAIDs on bone and tendon [10–27]. Little is known about their impact on tendon-to-bone healing [11, 25, 28–30]. Our results clearly show a significant decrease of intraarticular PGE2 concentration after Celecoxib treatment. Furthermore, new bone formation and tendon-to-bone healing may be impeded by selective COX-2 inhibitor treatment after ACL reconstruction. Thus, reluctant use is recommended until further studies complete clinical recommendations.
